# The outbreak that changed meat and poultry inspection systems worldwide

**DOI:** 10.1093/af/vfy017

**Published:** 2018-09-19

**Authors:** Elsa A Murano, H Russell Cross, Penny K Riggs

**Affiliations:** 1 Borlaug Institute, Texas A&M University, College Station, TX; 2 Department of Animal Science, Texas A&M University, College Station, TX

**Keywords:** adulterant, *Escherichia coli* O157:H7, Hazard Analysis and Critical Control Points, Jack-in-the Box, risk-based inspection

**Implications**

- In the early 1990s, meat and poultry inspection was slowly evolving away from a visual inspection system to one based on risk.
- Implementation of Hazard Analysis and Critical Control Points was moving toward the “raw” side of the system, on a voluntary, but slow basis.
- In 1992, the United States had not implemented standardized systems to train government or industry personnel on the use of Hazard Analysis and Critical Control Points.
- The 1993 Jack-in-the Box outbreak changed it all!

## Introduction

In the course of history, one of the best recognized constants is that change happens most often and most rapidly in response to a dire situation. Even when efforts to introduce improvements have been ongoing for quite a while, it is not until an emergency or a crisis develops that people are ready to accept change to resolve the problem at hand. Such has been the case with meat and poultry safety. Many in the community, whether regulators, the industry, researchers, or advocates would agree that the most significant and impactful such event was the 1993 outbreak of foodborne illness associated with the Jack-in-the-Box restaurant chain. According to the Washington Department of Health (**WDOH**), where the outbreak was first reported, a cluster of children were suffering from hemolytic uremic syndrome (**HUS**) caused by infection with *Escherichia coli* O157:H7, a little-known bacterial pathogen (CDC, 1993). The investigation quickly determined that the regular-sized hamburger patties and jumbo patties produced by Vons’ Companies of California and sold to the public by Jack-in-the Box were the source of the organism. The outbreak resulted in more than 600 illnesses with four children dead from having consumed undercooked hamburgers. To say that this was a scandal is an understatement. This situation was so dire that the outbreak was discussed as an agenda item during President Bill Clinton’s first cabinet meeting in 1993, after his inauguration as the 42nd President of the United States (Cross, Personal Communication, 2018). Many things changed following that meeting, culminating in a regulatory mandate by USDA’s Food Safety & Inspection Service (**FSIS**) for industry to implement the Hazard Analysis and Critical Control Point (**HACCP**) system at every federally inspected meat and poultry slaughter and processing plant in the United States. However, it may come as a surprise to some that the foundation for changes in food safety processes had already been in the works the year preceding the outbreak. A review of the events that took place in 1992 and how those activities had a direct impact on the events to come is justified.

## Prequel-1992

In January, 1992, Dr. Russell Cross became the Administrator of FSIS under President George H.W. Bush. With the assistance of outstanding personnel at the Agency, he began a total restructuring of FSIS that would allow it to move away from the “organoleptic only” type of inspection system that relied on sight and smell to more risk-based approaches (Cross, Personal Communication, 2018). Many of these moves met with strong resistance from the meat inspection union as well as from several consumer activist groups. What evolved over the next 12 mo became the Agency’s plan titled “War on Pathogens.” Elements of the plan included the formation of a HACCP Operations Task Force, and the implementation of a baseline testing program for steers and heifers, which had been recommended by the National Academy of Science in 1985 (NAS, 1985). The plan also prioritized public health over economic protection, the sharing of responsibilities between producers, government regulators, and consumers, and a restructuring of FSIS to better address food safety issues. The accompanying article in this issue of Animal Frontiers by Weinroth et al. (2018) does an excellent job of describing the evolution of HACCP implementation, thus will not be repeated here. The “War on Pathogens” plan was ready to be submitted to President Bush in November, 1992 for his approval, but the Presidential election in which Bill Clinton defeated the incumbent Bush disrupted those plans.

## The Outbreak

Fast-forwarding to 1993 and the Jack-in-the-Box outbreak, Administrator Cross received a phone call at midnight and was instructed to meet new USDA Secretary Michael Espy at Andrews Air Force Base at 6 a.m. the following morning, so they could fly together on Air Force II. The purpose of the trip would be to meet with the governor of the state of Washington and testify in front of the state senate about the situation (Cross, Personal Communication, 2018). Administrator Cross had not yet met Secretary Espy. It was during that flight that Cross was able to brief the new Secretary on the outbreak, actions taken by FSIS in response, and the details associated with the “War on Pathogens” plan. Upon arrival at the governor’s office building, Secretary Espy and Administrator Cross found thousands of concerned citizens on hand. Tempers were riding so high that Cross and Espy had to have an escort to get to the governor’s office, where much of their discussion centered around the “War on Pathogens” plan.

**Figure 1.**
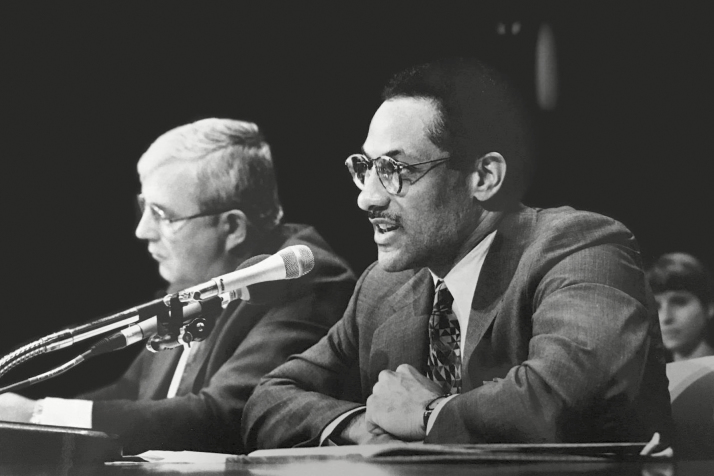
Administrator Cross and Secretary Espy testifying to the U.S. Congress.

On the return trip, as Secretary Espy and Cross began to discuss their preparations for testifying about the “War on Pathogens” plan to the U.S. Senate and House Agriculture Committees (Figure 1—Administrator Cross and Secretary Espy testifying to U.S. Congress), Espy asked Cross a key question: “How many of the federally inspected meat & poultry plants are voluntarily practicing HACCP on raw products?” After gathering the information, Cross told the Secretary that no more than 300 out of the 7,000-plus plants were doing so, recommending to the Secretary that HACCP be mandated for all federally inspected meat and poultry plants in the United States. At the time of his recommendation, Cross knew that neither USDA nor the industry was currently prepared to complete this task (Cross, Personal Communication, 2018).

In retrospect, the outbreak and the death of four children triggered several events which began moving quickly. Among these were the enforcement of zero fecal tolerance for beef carcasses, culminating with the decision to mandate HACCP in May, 1993. By the next year, at the end of his 2-yr federal assignment, Cross returned to Texas A&M University, where he was a professor in the Department of Animal Science. Secretary Espy then named Michael Taylor as the new FSIS Administrator. Taylor did not waste much time, declaring *E. coli* O157:H7 to be an adulterant in raw ground beef later that year. This action triggered a sampling program to test for the organism in federally inspected establishments and in retail stores, with product found to be contaminated being immediately declared “adulterated.” It was 1995 before the new Pathogen Reduction-HACCP Systems Rule would be proposed and then finalized in 1996 as the most comprehensive rule ever published by USDA on meat and poultry inspection (Federal Register, 1996). In addition to domestic meat products, the rule required that all countries that sell meat and poultry to the United States would need to have an “equivalent” system of inspection, such as HACCP. Certainly, the best way for countries to comply would be to simply mandate HACCP as well, which most did, demonstrating the worldwide impact of the PR-HACCP rule. As a result of the rule, the meat and poultry industry began the process of gearing up to implement HACCP in their operations, and to examine the use of pathogen interventions such as hot water and lactic acid rinses of carcasses to minimize the risk of contamination from the newly declared adulterant, *E. coli* O157:H7.

## International HACCP Alliance

As a relevant side-note, in the Spring of 1994, as Dr. Cross was traveling back to College Station, TX to resume his university position, he had a memorable conversation with Rosemary Mucklow, the then Executive Director of the National Meat Association (now part of North American Meat Institute). Cross and Mucklow both knew the challenges facing the USDA and the meat industry as they prepared to implement the new rule. They were both aware of the lack of HACCP-trained personnel within USDA, the meat sector or the universities. They were also aware of the lack of any standardized methodology to provide accreditation for “HACCP Training.” This discussion culminated in the creation of what is now known as the International HACCP Alliance, established and led by Cross in 1994 as a 501c3 organization charged with providing accreditation for HACCP training. The International HACCP Alliance has been very active ever since. The International HACCP Alliance is still housed in the Department of Animal Science at Texas A&M University, with Dr. Kerri Gehring serving as its CEO since 1997 (Jackson et al., 1996).

## Post PR-HACCP Rule

**Figure 2.**
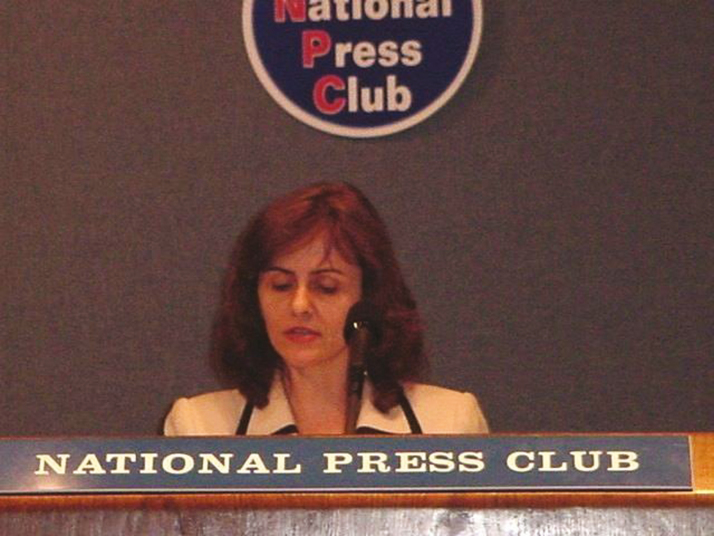
Undersecretary Murano speaks to reporters about food safety activities.

It is undeniable that the work that went into the “War on Pathogens” plan was the preamble, and the necessary foundation for the development of the PR-HACCP rule and for all the improvements in meat and poultry safety that followed. According to the Centers for Disease Control and Prevention, the relative rate of *E. coli* O157:H7 infections decreased to less than 0.9 by 2001, compared with 2.4, which was the 1996 to 1998 baseline established as a result of implementation of the PR-HACCP rule (CDC, 2008). This period was an exciting time for food safety, as it demonstrated the strength of science-based policies in improving public health. However, the celebration was somewhat short-lived. Just 5 yr later, in 2002, an outbreak involving ground beef produced at a Conagra meat processing plant in Greeley, Colorado, culminated in the recall of 19 million pounds of product, the second largest recall of ground beef at that time (MMWR, 2002). FSIS was in its first year under the leadership of a new Undersecretary for Food Safety, Dr. Elsa Murano, who quickly mobilized the Agency to determine what had gone wrong (Figure 2). After all, HACCP and testing for *E. coli* O157:H7 in product were operational in all meat and poultry processing plants. A scientist and university professor from Texas A&M University herself, Dr. Murano and her outstanding team of professionals at FSIS quickly ascertained four possible root causes. First, the inspector’s union contract had not allowed for meat inspectors to conduct the additional tasks that would be required for an in-depth analysis of HACCP plans in each plant. Their main function had only been to verify the existence of HACCP plans, not their scientific soundness. Second, many industry operations did not consider *E. coli* O157:H7 a hazard “reasonably likely to occur” as part of their hazard analysis; thus, suppliers of beef trimmings were not held accountable by grinders for their ability to control contamination with this hazard. Third, a culture of overreliance on microbial testing had emerged, with some groups insisting that testing final product for the presence of the pathogen would be enough to prove its safety. The problem with this approach is that pathogens are not evenly distributed throughout product, thus not finding *E. coli* O157:H7 in a 25-g sample does not guarantee that it is not present in the rest of the lot. In other words, “absence of evidence is not evidence of absence.” Fourth, some in the community were not willing to accept the impossibility of attaining zero-risk in raw foods of animal origin without further processing. Thus, application of effective technologies such as food irradiation was deemed politically unacceptable (Murano, Personal Communication, 2018).

The Agency rolled up its sleeves and engaged in the training of its inspectors in the science of HACCP, forming teams of HACCP auditors to ensure that plans were not only in effect, but that they had been developed in a science-based manner. The Agency also mandated a reassessment of each plant’s hazard analysis, with special directives aimed at holding suppliers to beef grinding operations accountable for controlling the risk of contamination with the pathogen. A new chapter of professional cooperation between the Agency and the industry was forged, with FSIS encouraging plants to test and hold their product from shipping until the tests were completed. As was once stated by Dr. Dane Bernard, former Vice President of Food Safety and Quality at Keystone Foods, “the Agency believed our negatives, not just our positives.” This attitude resulted in increased testing for the pathogen by industry as a verification of control through the sound implementation of HACCP, rather than the previous emphasis on end-product testing as the only proof of safety. In addition, the meat industry came together to proactively continue their vigilance in doing all it could to mitigate risk through the establishment of the Beef Industry Food Safety Council (**BIFSCO**). This group, represented by some of the largest meat companies in the United States, still meets to this day, engaging in the development of science-based strategies and supporting research to prevent pathogen contamination of beef products. As a result of all these efforts, by the end of Dr. Murano’s service as undersecretary in December of 2004, the number of foodborne illnesses due to *E. coli* O157:H7 had decreased by 46%, achieving CDC’s Healthy People 2010 goal for this pathogen 6 yr ahead of schedule. Massive recalls of ground beef also decreased from a high of 21 cases involving large volume of product in 2000, to very few and very small recalls by 2004.

As the opening paragraph of this article attests, the work conducted in the early 1990s certainly served to lay the groundwork for the system that would serve to protect public health for years to come. The 2002 Conagra outbreak simply affirmed the fact that a great system is only made great if it is effectively implemented and enforced. About 5 yr later, unfortunately, this lesson had to be relearned through an outbreak in 2007 due to consumption of ground beef sold by Topps Meats. In that situation, the source was identified as trimmings the company obtained from Rancher’s Beef Ltd. in Alberta, Canada. Almost 100 illnesses were reported in the United States and Canada, culminating in the second largest recall of ground beef in U.S. history, 21.7 million pounds. This was second only to a recall harkening back to the pre-HACCP rule era in 1997, when Hudson Foods Company recalled 25 million pounds of product due to contamination with *E. coli* O157:H7. According to an audit conducted of Canada’s meat, poultry, and egg products inspection system in 2006, a year prior to the Topps Meats outbreak, FSIS found that 15 out of 21 establishments in that country had deficiencies in the implementation, corrective actions, verification, and/or recordkeeping parts of their HACCP plans. In fact, in seven establishments, verification and validation of HACCP were not performed properly. Clearly, these were red flags, and in the Spring of 2007, before the outbreak took place, an audit of the Canadian system showed that when total coliforms and generic *E. coli* counts exceeded the limits, the Canadian Food Inspection Agency (**CFIA**) took no action. Worse yet, in one establishment, there had been no visit by an inspector from the CFIA during the second shift for a 2-mo period. In the aftermath of the outbreak, FSIS de-listed Rancher’s Beef Ltd., otherwise known as “Establishment 630.” A special audit of Canada was then conducted by FSIS in November, 2007, along with the announcement by the new undersecretary, Dr. Richard Raymond, that it would begin increased product testing of Canadian products shipped to the United States. Obviously, a lack of enforcement by CFIA was at the root of the problem, with Canada facing the need to ramp up enforcement of its rules, not to mention its commitment to equivalence to the U.S. government. The Canadian government now has in place testing programs for Shiga toxin-producing *E. coli* in beef manufacturing trimmings destined for export to the United States, a practice it had not implemented before. As one possible lesson to learn, perhaps FSIS should take a new look at how it defines “equivalence” when it comes to imported beef, and to require more tangible proof during its audits than can be gleaned from documents and on-site reviews, to demonstrate that the level of protection against food hazards is the same as in the United States.

## Conclusions

The Spanish philosopher and poet Jorge Santayana once said, “those who cannot remember the past are condemned to repeat it.” This year marks the 25th anniversary of the Jack-in-the-Box outbreak. This event changed the history of food safety in our country, if not the world, which most would recognize as a necessary event for the changes that followed to be accepted. As much as we have tried never to repeat this type of incident, we have presented examples of instances where we failed to learn from it, resulting in additional outbreaks of foodborne illness. In deference to Mr. Santayana, we would like to offer one additional recommendation, that is, to continue to improve our food safety systems even in the absence of a crisis. We recommend engagement in “what if” scenarios in order to anticipate and thereby prevent outbreaks as much as science will allow, and to not wait for the situation to demand action. Lastly, we also suggest to the next generation of leaders that they must avail themselves of the vast experience that surrounds them in terms of government, industry, and university leaders who lived through these outbreaks so that they can, in effect, learn about the past and thus avoid repeating it.

## About the Authors

Dr. H. Russell Cross is a Professor in the Department of Animal Science in the College of Agriculture and Life Sciences at Texas A&M University. He most recently served as Head of the Department of Animal Science and as Executive Vice President for Operations and Chief of Staff to Texas A&M President Elsa Murano. Prior, he served as Deputy Vice Chancellor and Associate Dean for Agriculture and Life Sciences. His service in government has included the role of Administrator of USDA’s Food Safety and Inspection Service under Presidents Bush (41) and Clinton. Within the private sector, Dr. Cross was CEO and Chairman of Future Beef Operations, L.L.C., and Director of Food Safety for IDEXX Laboratories. Within National Beef Packing Co., Dr. Cross served as Executive Vice President of Food Safety/Government and Industry Affairs.

A graduate of Florida International University (B.S. in Biological Sciences) and Virginia Tech (M.S. in Anaerobic Microbiology and PhD in Food Science & Technology), Dr. Murano began her career as an Assistant Professor in food microbiology at Iowa State University in 1990. In 1995, she joined the faculty at Texas A&M University as Associate Professor, leading the university’s Center for Food Safety as Director. In 2001, Professor Murano was appointed by President George W. Bush to serve as Undersecretary for Food Safety at the U.S. Department of Agriculture, the highest ranking food safety official in the U.S. government. In 2005, Dr. Murano returned to Texas A&M, where she was appointed Dean and Vice Chancellor of Agriculture and Life Sciences, the largest such program in the nation. In 2008, Dr. Murano became the first woman, and first Hispanic president of Texas A&M University. Since June of 2012, Dr. Murano has been serving as Director of the Norman E. Borlaug Institute for International Agriculture.

Dr. Penny Riggs is an Associate Professor in the Department of Animal Science at Texas A&M University. She has broad, multidisciplinary interest in the role of animal agriculture and biotechnology for food and nutritional security. She has chaired the Council of Principal Investigators at Texas A&M, and the Public Policy Committee of the American Society of Animal Science. Current research interests include genetic parameters that regulate or determine aspects of beef quality and nutritional value, skeletal muscle growth, and developmental processes. She completed B.S. and M.S. degrees in Biology and Cytogenetics at Purdue University, and a Ph.D. in the interdisciplinary program in Genetics at Texas A&M University. Following postdoctoral research at NASA L.B. Johnson Space Center, and M.D. Anderson Cancer Center, she returned to Texas A&M University as a faculty member.
